# Apathy, Beta-amyloid Burden and Cognitive Decline in Parkinson’s Disease Patients

**DOI:** 10.1192/j.eurpsy.2025.1664

**Published:** 2025-08-26

**Authors:** R. Fernández-Fernández, R. Rodríguez-Rojas, C. Trompeta, B. Fernández-Rodríguez, G. Lahera, C. Gasca-Salas

**Affiliations:** 1 HM CINAC (Centro Integral de Neurociencias Abarca Campal), Hospital Universitario HM Puerta del Sur, HM Hospitales; 2 Instituto de Investigación Sanitaria HM Hospitales, Madrid; 3 Hospital Universitario Infanta Cristina, Parla; 4 PhD Program in Health Sciences, University of Alcalá de Henares., Alcalá de Henares; 5 Network Center for Biomedical Research on Neurodegenerative Diseases (CIBERNED), Instituto Carlos III; 6 PhD Program in Neuroscience, Cajal Institute, Autónoma de Madrid University, Madrid; 7 Department of Medicine and Medical Specialities, University of Alcala, Alcalá de Henares; 8 Ramón y Cajal Institute of Sanitary Research (IRYCIS), Madrid; 9 Psychiatry Service, Center for Biomedical Research in the Mental Health Network, University Hospital Príncipe de Asturias, Alcalá de Henares; 10 University CEU-San Pablo, Madrid, Spain

## Abstract

**Introduction:**

Apathy is a common non-motor symptom in Parkinson’s disease (PD), and its presence constitutes a risk factor for the development of cognitive impairment in this population (Burchill et al. Lancet Reg Health Eur 2024; 39:100870). β-amyloidopathy has been associated to shorter interval to dementia in PD and may also be a determinant of apathy.

**Objectives:**

We aimed to investigate β-amyloid burden in non-demented PD patients based on the presence or absence of apathy, and how both factors influence the rate of progression to mild cognitive impairment or dementia over a 3-year period.

**Methods:**

Forty-eight PD patients underwent clinical and comprehensive neuropsychological evaluation, as well as [18F]-flutemetamol positron emission tomography. They were classified as apathetic (n=22) or non-apathetic (n=26) based on their score on the Starkstein Apathy Scale. Brain imaging analysis was conducted using the SPM software package.

**Results:**

We found statistically significant differences in disease duration when comparing clinical and demographic variables. Upon neuropsychological evaluation, apathetic patients performed significantly worse in attention domain (Digit Span and Trail Making Test A), executive function (Stroop Word-Colour Test and Trail Making Test B) and verbal memory (CERAD Total Word Recall). At follow up, 47.4% of apathetic patients progressed to dementia or MCI, compared to 12% of non-apathetic patients (χ² = 6.81, p <0.05). Brain imaging analysis revealed higher β-amyloid deposition in several cortical areas in apathetic PD patients (adjusted for disease duration and global composite cognitive z-scores).

**Conclusions:**

The presence of apathy in PD patients is associated with greater cortical β-amyloidopathy and indicates a higher conversion rate to a worse cognitive diagnosis within a 3-year period.

**Disclosure of Interest:**

None Declared

